# Diagnostic dilemmas in a girl with acute glomerulonephritis: Answers

**DOI:** 10.1007/s00467-017-3626-3

**Published:** 2017-03-09

**Authors:** Farah A. Falix, Michiel J. S. Oosterveld, Sandrine Florquin, Jaap W. Groothoff, Antonia H. M. Bouts

**Affiliations:** 1 Pediatric Nephrology Department of the Emma Children’s Hospital, Academic Medical Center, Amsterdam, Noord-Holland Netherlands; 2 Pathology Department, Academic Medical Center, Amsterdam, Noord-Holland Netherlands

**Keywords:** Rapidly progressive glomerulonephritis, Post-infectious glomerulonephritis, C3 glomerulonephritis

## Answers

**Fig. 1:**
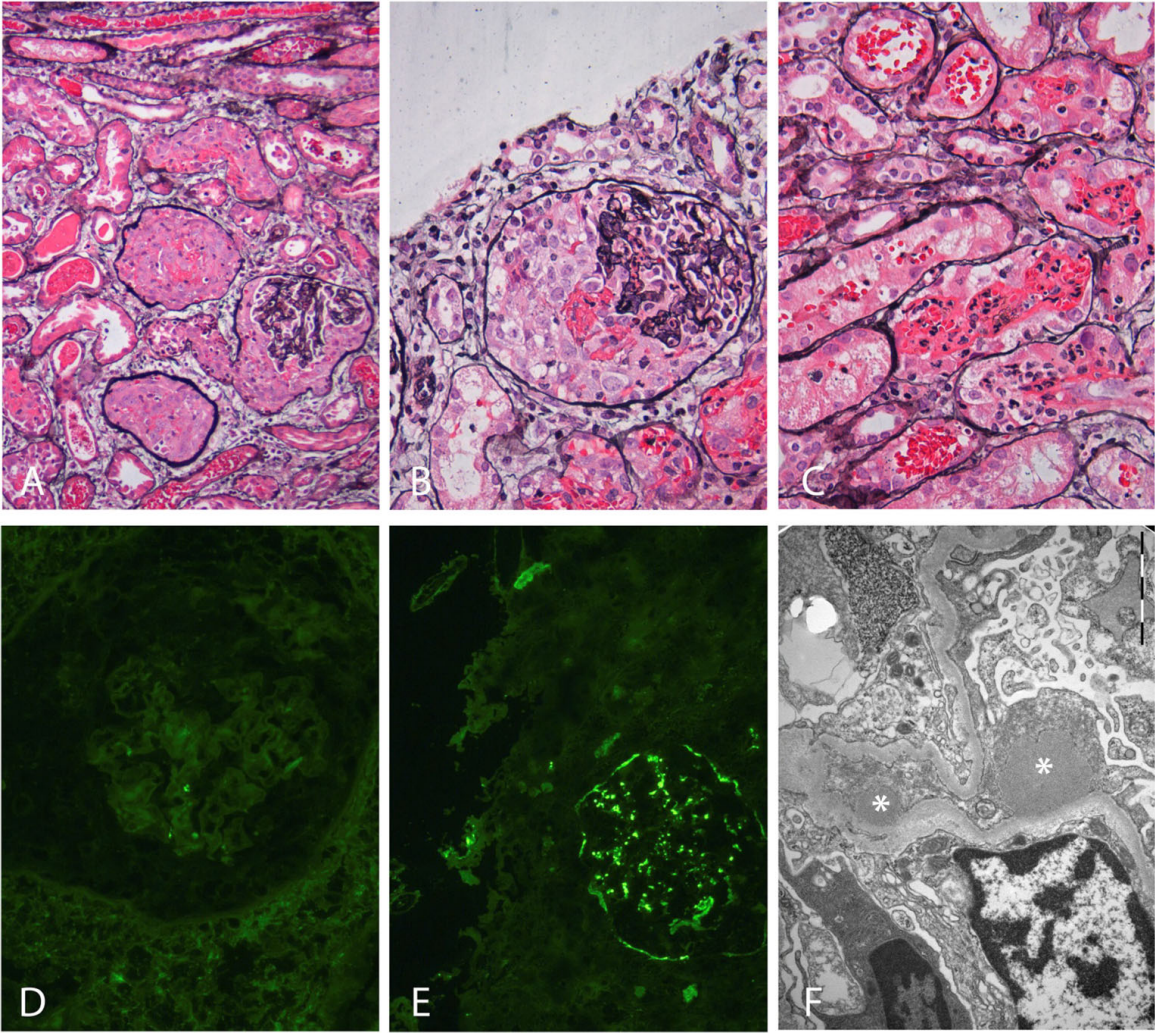
Light microscopy, immunofluorescence, and electron microscopy findings in the renal biopsy. **a** Light microscopy shows three crescentic glomeruli (100% crescents in the whole biopsy), together with some interstitial edema and dilated tubules (silver staining, ×10). No signs of chronicity. **b** Higher magnification of a crescentic glomerulus with some endocapillary hypercellularity due to granulocyte infiltration (silver staining, ×20). **c** Light microcopy shows dilated tubules filled with erythrocytes and granulocytes (silver staining, ×20). **d** Negative immunofluorescent staining of a glomerulus for IgG (×20). **e** C3c immunofluorescent staining showing coarse granular deposits along the capillary walls (2+), the so-called “starry-sky” pattern (×20). **f** Electron microscopy showing large sub-epithelial electron-dense deposits (“humps”) marked with *asterisks* associated with effacement of the podocytes
  Intermittent hemodialysis was started on the fourth day after admission. AST and anti-DNAse B titer were still not available, nor were the bacterial and viral serology investigations. As renal biopsy findings were consistent with both PIGN and C3GN, a diagnosis of C3GN due to an intrinsic complement defect, triggered by an infection, could not be ruled out, despite the normal complement C3 level. In view of the severity of the clinical course, the biopsy findings with 100% crescents and recent publications reporting promising results of the complement inhibitor eculizumab for C3GN [9–11], a single dose of eculizumab (375 mg/m^2^ body surface area) was administered intravenously awaiting further results. Complement cascade diagnostic investigations had been undertaken before eculizumab administration to prevent difficulties in interpretation of the results. Over the following days, intermittent hemodialysis was continued. The AST returned highly positive (1,600 U/ml), as did the anti-DNAse B titer (>1,600 U/ml), indicative of a recent streptococcal infection. Mycoplasma and hepatitis B/C serology were negative. Complement cascade analysis showed mild complement alternative pathway route activation (114%; reference value: 40–110%), with elevated alternative complement byproduct C3d (4.8%; reference value: 0.5–3.1%) and normal C5b-9 (<1,2 AE/ml; reference value:<4 AE/ml). C3 nephritic factor was negative, as were ANA, ANCA, and anti-GBM antibodies. From the sixth day after admission onward, renal function improved rapidly and hemodialysis treatment was discontinued. The patient was discharged 11 days after admission, with a serum creatinine level of 106 μmol/l (1.2 mg/dl) and without medication. At last follow-up, 2 months after presentation, renal function had completely recovered (creatinine 42 μmol/l (0.5 mg/dl), eGFR according to Schwartz was 116 ml/min/1.73 m^2^), and proteinuria levels had declined significantly (protein to creatinine ratio: 55.2 mg/mmol Cr). This clinical course of rapid improvement favors the diagnosis of PIGN with RPGN presentation.

1. Based on the initial presentation of macroscopic hematuria, proteinuria, and renal failure following an episode of pharyngitis with a typical time lag of 2 weeks, the differential diagnosis consists primarily of acute post-infectious glomerulonephritis (PIGN), the first presentation of either immune complex, or complement-mediated membranoproliferative nephritis (MPGN), or IgA nephropathy (IgAN) triggered by an infection. Less likely, but not excluded, are ANCA vasculitis, lupus nephritis, anti-glomerular basement (GBM) glomerulonephritis, and toxicity-/medication-induced glomerulonephritis. The young age and lack of sinusitis or pulmonary symptoms make ANCA vasculitis less likely and there were neither physical nor anamnestic signs of systemic lupus erythematosus (SLE). Macroscopic hematuria and nephrotic proteinuria are less consistent with a toxic cause of acute kidney injury. Given the clinical course, the patient had suspected rapidly progressive glomerulonephritis (RPGN).
2. Additional laboratory investigations should be aimed at possible causes of RPGN [1]. The few available studies on pediatric RPGN report that it is caused in over 80% by a form of immune-complex-mediated glomerulonephritis, with MPGN, Henoch–Schönlein purpura (HSP)/IgA nephropathy and PIGN as the most frequent underlying causes [2–5]. Lupus nephritis, C3 glomerulonephritis (C3GN), and ANCA vasculitis are more infrequent causes of pediatric RPGN [2–5]. In our patient, additional serum measurements included anti-streptolysin titer (AST) and anti-DNAse B titer, mycoplasma, hepatitis B and C serology, ANA, anti-PR3 ANCA, anti-MPO ANCA, anti-GBM measurements, and a full complement cascade for assessment of both the classical and alternative pathways (C3, C4, C3d, AP50, CH50, C3 nephritic factor, C5b-9).
3. On the day after admission, the patient developed oliguria and a steep rise in creatinine level, indicating a rapid progression of renal failure. Although there was a high degree of suspicion regarding PIGN, serum complement C3 and C4 levels and AST and anti-DNAse B titer were not yet available. Generally, a renal biopsy is not mandatory when PIGN is suspected. However, certain features atypical of PIGN should prompt a biopsy. These include RPGN, extra-renal manifestations, a patient’s age <2 years, persistent gross hematuria, hypertension or nephritic syndrome, and hypocomplementemia lasting >6 weeks [6]. In view of the rapid course of the acute kidney injury in our patient, a renal biopsy was planned for the next available appointment, which was the next day.
4. There is little evidence regarding the treatment of RPGN in children and existing guidelines are based on studies in adults, in whom the underlying cause of the glomerulonephritis is usually different. For instance, adults have a higher likelihood of ANCA vasculitis [1]. Nevertheless, there is a general consensus that methylprednisolone pulse therapy with or without cyclophosphamide, is the first therapeutic regimen in RPGN [2–5, 7]. In our patient, we refrained from immunosuppressive treatment before a biopsy had been obtained. At the time, there was a high degree of suspicion regarding either PIGN or MPGN (immune complex- or complement-mediated). Methylprednisolone pulse therapy was not started, as there is a lack of evidence for a beneficial effect of immunosuppressive therapy in PIGN [7, 8]. Also, we feared that high doses of steroids might influence the renal biopsy. “Blind” administration of eculizumab was also considered but rejected for lack of a diagnosis and in view of its high costs. Supportive treatment consisting of fluid restriction, diuretics, and antibiotics was continued, awaiting the serum complement results.
5. In our patient, the renal biopsy and serum complement results imposed a diagnostic dilemma, which is described below. On the third day of admission, a Monday, serum complement measurements were found to be normal (C3: 1.15 g/l, reference value 0.9–1.8 g/l; C4: 0.43 g/l; reference value 0.1–0.49 g/l). AST and anti-DNAse B titer were not yet available. Creatinine levels continued to rise to 640 μmol/l (7.2 mg/dl) and oliguria persisted. Because of the clinical picture of RPGN, together with the absence of hypocomplementemia characteristic of PIGN, this initial diagnosis was considered less likely, as was a complement-mediated MPGN. A renal biopsy was performed on the same day, combined with the placement of a central venous line for hemodialysis. Methylprednisolone pulse therapy (three daily pulses of 500 mg/m^2^ body surface area) was started immediately after the renal biopsy. Histopathological evaluation of the renal biopsy showed crescentic glomerulonephritis in all 25 glomeruli (100% crescents), together with some endocapillary hypercellularity due to granulocyte infiltration. All crescents were cellular, with no signs of chronicity (Fig. 1a, b). There was also mild interstitial edema and tubules were dilated and filled with erythrocytes and granulocytes (Fig. 1c). Immunofluorescence was negative for IgG (Fig. 1d), IgM, IgA, kappa, lambda, and C1q, but revealed coarse granular C3c deposits along the capillary walls (2+), the so-called “starry-sky” pattern (Fig. 1e). C4d staining was negative and C5b9 staining was similar to C3c. Electron microscopy showed several (subepithelial) humps (Fig. 1f) and tiny subendothelial electron dense deposits. Intramembranous garland-like electron dense deposits compatible with dense deposit disease (DDD) were not observed. These findings could be associated with both C3GN and severe PIGN.

## Discussion

Post-infectious glomerulonephritis is an immunologically mediated glomerular injury triggered by an infection. In children, >95% of cases are caused by streptococcal infections. Poststreptococcal glomerulonephritis (PSGN), induced by “nephritogenic” group A streptococcal pharyngitis or skin infections, is a classic example of PIGN with diffuse proliferative and exudative glomerular histology, dominant C3 staining, some IgG deposition, and subepithelial hump-like deposits [6, 12]. Typical PSGN usually presents with acute nephritic syndrome of variable severity, transient serum C3 hypocomplementemia (for 4–8 weeks) and generally resolves without complications within a short period [6, 8, 13]. Most children and young adults with PIGN have an excellent prognosis and >90% regain normal renal function at short-term follow-up. Poor prognostic factors include nephrotic syndrome, acute kidney injury requiring dialysis, large and fibrous crescents, and signs of chronicity in the renal biopsy [2, 6, 8, 13, 14]. The few available long-term follow-up studies suggest that PSGN might not be entirely a benign disease, with persistent non-nephrotic proteinuria or hematuria in some children and development of ESRD in <1% of patients [6, 14, 15]. Studies regarding pediatric RPGN or crescentic glomerulonephritis, report PIGN as the underlying cause in 7–50% of cases, with the highest incidence of PIGN in developing countries [2–5]. One study of 67 pediatric RPGN patients in Thailand reported progression to ESRD in 35% of patients at a median follow-up of 1 year [5]. In the more severely affected patients, those with >50% crescents on biopsy and all treated with methylprednisolone pulse therapy, 11 out of 19 patients (57%) proceeded to ESRD. In this study, RPGN was ascribed to PIGN in 50% of cases, and 16.7% of ESRD patients had PIGN as an underlying etiology. The study concluded that patients with RPGN from PIGN had a better renal outcome than patients with RPGN of other etiologies, with the worst prognosis for immune-complex-mediated glomerulonephritis and lupus nephritis (54.1% and 29.2% of ESRD patients respectively) [5]. Other pediatric studies report incidences of CKD/ESRD of 0–31% for PIGN presenting with RPGN, with the highest incidence of ESRD in developing countries [2–4, 7]. One explanation for this higher prevalence of ESRD in developing countries may be delayed referral, as signs of chronicity were often reported in the biopsies [3, 5].

In our patient, the high AST and anti-DNAse B titers combined with the renal biopsy findings and favorable outcome despite the presence of 100% crescents on biopsy, strongly favor PIGN as the underlying cause of RPGN. The finding of (repeatedly) normal complement C3 levels in our patient is unusual, but has been reported previously, with incidences ranging from 5 to 36% in patients with PIGN requiring a renal biopsy [7, 13, 16]. In the study by Wong et al., normal complement C3 levels were reported in 9 out of 25 patients with severe PIGN, 4 of whom required hemodialysis. One of these patients had 100% crescents in the renal biopsy and similar to the course of our patient, this patient had a favorable outcome with a creatinine level of 87 μmol/l (0.9 mg/dl) at the age of 11.7 years after almost 8 years of follow-up [7].

Another remarkable biopsy finding in our patient was the dominance of C3 staining on immunofluorescence and a complete lack of IgG deposition. This finding can be associated with a late biopsy during the course of PIGN, as IgG depositions may decline during the course of the disease [14, 15]. Generally, dominant C3 staining is more consistent with C3 glomerulopathy (C3G) [9, 17, 18]. C3G is a relatively new term describing a spectrum of glomerular patterns of injury that is associated with complement alternative pathway dysregulation with predominant C3 deposition and absent or negligible immunoglobulin staining in renal biopsies. C3G can be histologically divided into DDD and C3GN, depending on the pattern and location of the deposits. Humps can be found in both DDD and C3GN [9, 18]. More than 90% of patients with C3G show depressed serum complement C3 levels, but levels may vary during the course of the disease [9, 18], and genetic mutations in complement alternative pathway regulation are increasingly reported [18, 19]. In our patient, the immunofluorescence and electron microscopy findings (coarse and granular C3 pattern, IgG negative, subepithelial deposits (humps), a few nonlinear subendothelial electron-dense deposits) were compatible with both PIGN and C3GN, making it difficult to establish the appropriate therapeutic approach. Therefore, considering the severe clinical presentation, a therapy regimen based on both diagnoses was chosen, despite the lack of evidence for beneficial effects of eculizumab on C3G [19]. Interestingly, a recent study retrospectively describes and re-defines biopsy findings in 33 pediatric patients initially diagnosed with PIGN and hypothesizes that PIGN and C3G might define a disease spectrum [17]. The authors re-define PIGN and C3G subgroups based on C3 and IgG staining patterns and the presence and location of electron-dense deposits as follows: a PIGN group (*n* = 25) with subepithelial deposits and positive C3 staining with IgG staining present (group A) or absent (group B), a C3G group (*n* = 8) based on dominant C3 staining without subepithelial deposits regardless of IgG staining (subgroup C) or dominant C3 staining with intramembranous dense deposits and absent IgG staining (subgroup D). The newly defined PIGN patients had a more favorable clinical outcome (85% complete recovery), compared with the newly defined C3G group (66% and 25% complete recovery for groups C and D respectively). The authors conclude that patients with C3G tended to have a milder and more insidious clinical presentation, but a worse prognosis. It is interesting to note that in the reported study, only 1 of the 23 PIGN patients with detailed clinical reports had a clinical profile similar to that of our patient (i.e. dominant C3, absent IgG, humps, and an absence of C3 hypocomplementemia) [17]. The above underscores the difficulty in interpreting the clinicopathological data in our patient. During follow-up, our patient showed normalization of the initially raised C3d levels and her serum C3 levels remained normal. Therefore, the diagnosis of C3GN was rejected and genetic screening for complement alternative pathway mutations (i.e., complement factor H and related proteins, complement factor I mutations) was not performed. It should be recognized that C3G may present following an infectious episode, often a streptococcal infection, and that subepithelial deposits may also be a feature of C3GN. Therefore, the presence of any atypical clinical or histological features in a case of apparent PIGN should raise the suspicion of C3GN and warrant alternative complement pathway investigations, including mutational screening.

In summary, our case shows that PIGN can have a favorable course, despite a presentation as RPGN with massive crescent formation on biopsy and acute kidney injury requiring temporary dialysis. Involvement of the alternative complement pathway route, as observed on renal biopsy, can occur in the absence of a decreased plasma C3 level. Whether the combined administration of methylprednisolone and eculizumab positively influenced the course of disease might be a subject for further studies.

## Abbreviations

PSGN: Poststreptococcal glomerulonephritis
PIGN: Post-infectious glomerulonephritis
RPGN: Rapidly progressive glomerulonephritis
MPGN: Membranoproliferative glomerulonephritis
GBM: Glomerular basement membrane
IgAN: IgA nephropathy
HSP: Henoch–Schönlein purpura
AST: Anti-streptolysin titer
C3G: C3 glomerulopathy
C3GN: C3 glomerulonephritis
